# Effect of UV Top Coating Microcapsules on the Coating Properties of Fiberboard Surfaces

**DOI:** 10.3390/polym16152098

**Published:** 2024-07-23

**Authors:** Yuming Zou, Yongxin Xia, Xiaoxing Yan

**Affiliations:** 1 Co-Innovation Center of Efficient Processing and Utilization of Forest Resources, Nanjing Forestry University, Nanjing 210037, China; zou_yuming@njfu.edu.cn (Y.Z.); xiayongxin@njfu.edu.cn (Y.X.); 2 College of Furnishings and Industrial Design, Nanjing Forestry University, Nanjing 210037, China

**Keywords:** microcapsules, UV coating, self-healing, fiberboard

## Abstract

The commonly used ultraviolet ray (UV) curing coatings have the characteristics of fast curing speed, high hardness, strong abrasion resistance, etc. However, the self-healing properties of UV coatings after being damaged still need to be improved. Self-healing microcapsules can alleviate this problem. The UV top coating itself has good properties, so it can be directly chosen as the core material of microcapsules. UV top coating microcapsules can be added to the UV top coating to increase the self-healing properties of the UV coating to achieve the purpose of better protection of the UV coating and fiberboards. UV top coating microcapsules were prepared and added in different contents to characterize the effect on the physical, chemical, and self-healing properties of the UV coating on a fiberboard surface. The 1#, 2#, and 3# UV top coating microcapsules that were prepared with emulsifier HLB values of 10.04, 10.88, and 11.72, respectively, were added to the UV top coating at contents of 2.0%, 4.0%, 6.0%, 8.0%, and 10.0%. The UV coatings were applied to the fiberboard using a method of two primers and two top coatings, in which no microcapsule was added in the primer, and were tested and analyzed. The results showed that when the content of microcapsules was greater than 6.0%, close to 8.0%, the excessive density of microcapsules produced stacking and extrusion between the microcapsules. As a result, the core material could not flow out smoothly when part of the microcapsule was ruptured. The outflow of the core material was not efficiently utilized, thus leading to a decrease in the self-healing rate. The 2# UV top coating microcapsules of 4.0% made the UV coatings reach the self-healing rate of 26.41%. The self-healing rate of the UV coatings prepared with the 3# UV top coating microcapsules with 6.0% was up to 26.58%. The UV coatings prepared with the 1# UV top coating microcapsules of 6.0% had the highest self-healing rate among the three groups, up to 27.32%. The UV coatings of this group had the best comprehensive properties with a chromatic aberration Δ*E* of 4.08, a gloss of 1.10 GU, a reflectance of 17.13%, an adhesion grade of 3, a hardness of 3H, a grade 3 of impact resistance, and a roughness of 1.677 μm. An investigation of the UV coatings on fiberboard surfaces with the content of UV top coating microcapsules can provide support for the optimization of the self-healing properties of UV coatings and can also provide innovative ideas for the preparation of the self-healing coatings on fiberboard surfaces.

## 1. Introduction

Furniture connects closely with people’s production and living. According to different usage scenes and purposes, different materials are reasonably being used [1,2,3,4,5]. In recent years, fiberboard has been widely used in the area of furniture manufacturing because of its advantages such as high flatness, low price, and good surface treatment [6,7,8,9,10]. Fiberboard is usually made of wood fibers or other plant fibers processed by high temperature and high pressure. Furniture made of fiberboard is lightweight and has a smooth surface after coating [11,12]. Wood coatings are usually classified into six categories: unsaturated polyester coatings, polyurethane coatings, nitro coatings, acid-cured amino coatings, water-based coatings, and ultraviolet ray (UV)-cured coatings [13,14,15,16,17,18]. Among them, UV coatings are widely used because of the advantages of low processing cost and wide adaptability of substrates [19,20,21]. The most significant feature of a UV coating is that it can be cured quickly under UV irradiation [22,23]. UV coating is a type of environmentally friendly coating that is widely used [24,25]. Salca et al. [26] compared the properties of UV coatings and water-based coatings by investigating the gloss of black alder (*Alnus glutinosa* L.) samples coated with two types of coatings as a result of thermal exposure and artificial aging, as well as a chemical resistance of the coating samples to cold liquids. The results showed that the UV coating had higher gloss values than the water-based coating. The thermal exposure had a large effect on the gloss of the UV coating samples. The overall gloss of the samples decreased with artificial aging time. Alcohol could easily damage the UV coating. UV coatings have a wide range of applications in furniture finishing and other fields with excellent properties. However, UV coatings are easily affected by external factors in the process of their usage, such as impact, acid, and heat. The coating will unavoidably cause many microcracks, defects, etc. The appearance of the coating on the furniture surface and the stability of the internal structure of the furniture will be negatively affected, which will in turn decrease the extra value of the furniture. In order to alleviate the disadvantages of UV coating and to expand the multifunctionality of UV coatings, the modification of UV coatings has gradually become a hot spot in recent years.

There are many methods of modifying the coating on the surface of wood furniture, among which microencapsulation technology is more convenient and cheaper [27,28,29,30]. In the 1930s, researchers prepared a type of tiny spherical particle that consisted of a dense outer shell and different inner substances in the shell [31]. These particles were named microcapsules because their overall structure was similar to that of a capsule. And now, microcapsules have various structures [32,33,34,35]. With their wall–core structure, microcapsules can encapsulate a core substance with a certain function to achieve the corresponding effect [36,37]. For example, microcapsules were made of the core material substance with a healing effect through the selection of microcapsules and added to the coating. Then, the coating could be prepared to have a certain self-healing property. Microcracks always occur within the coating. This type of microscopic damage is difficult to detect. The presence of microcracks affects the various coating properties to a greater extent and shortens the coating duration. An inappropriate combination of wall materials and coatings can reduce the dispersibility of the microcapsules, such as melamine formaldehyde resin walls with acrylic water-based coatings. Through microencapsulation, the UV coating is highly self-healing through biomimetic biology that mimics the mechanism of biological scar healing [38]. The internal liquid outflow after the rupture of the microcapsule wall by external forces fills the microcracks so as to achieve the self-healing property of the coating. Recent studies on the oriented release of microcapsules are worth referring to, and in the future, the oriented release of core materials in microcapsules towards the microcracks of the coating is expected to be enabled [39]. Self-healing microcapsule technology has become a hot spot of applied research in recent years because of its high self-healing efficiency and low cost. Self-healing microcapsules have received wide attention because they can produce a good healing effect on a system in which they are located [40,41,42]. Research on the use of self-healing microcapsules to modify the properties of UV coatings has mainly focused on the healing of metal surfaces. Thakur et al. [43] researched the synthesis of microcapsules encapsulated by rosin-based epoxy resins and an imidoamine curing agent for self-healing coatings. The microcapsules were prepared by an in situ polymerization technique. A thermal degradation analysis was performed to assess the stability of the microcapsules. The self-healing coatings were applied on mild steels using rosin-based epoxy/imide containing 10 wt% and 20 wt% epoxy microcapsules as well as 10 wt% epoxy and 10 wt% curing agent microcapsules. Salt spray, adhesion, and tensile tests were performed on the coatings with and without microcapsules to determine the chemical resistance and mechanical strength of the self-healing coatings. The results showed that the microcapsules with an epoxy/imide coating containing both 10 wt% epoxy resin and 10 wt% curing agent performed the best. The application of the self-healing microcapsules in UV coatings for wood substrates is still to be explored. The application of UV coating microcapsules to increase the self-healing property of UV coatings on wood furniture surfaces is of practical significance.

A UV top coating itself was used as a core material, and simple, non-toxic, and odorless melamine formaldehyde resin was used as a wall material. Three types of UV top coating microcapsules were prepared; they were added to the UV top coating according to ratios and then coated on the fiberboard surface using a method of two primers and two top coatings without microcapsules in the primer. A test was performed to investigate the effect of the content of UV top coating microcapsules on the comprehensive properties of the coating on fiberboard surfaces.

## 2. Materials and Methods

### 2.1. Materials and Instruments

The size of fiberboards was 50 mm × 50 mm × 5 mm. The materials are shown in Table 1. The UV top coating included polyurethane acrylic resin, 1,6-hexanediol diacrylate, photoinitiator, functional filler, extinction powder, wax powder, defoamer, dispersant, anti-settling agent, etc. The solid content of the UV top coating was more than 98%. The test instruments are shown in Table 2; the lamp in the single-lamp curing machine was a UV mercury lamp with a light intensity of 80–120 W/cm^2^, and the wavelength range of ultraviolet radiation released was 250–420 nm.

### 2.2. Preparation Method of UV Top Coating Microcapsules

The details of material amounts of the 1#, 2#, and 3# UV top coating microcapsules are shown in Table 3. Using the in situ polymerization method of coating microcapsules, the capsule wall was generated by the reaction of melamine and formaldehyde. With the polymerization reaction, the molecular weight and chain length of the pre-polymer gradually increased. For water-soluble wall materials, the coating process involved the adsorption effect of the wall material from the continuous phase to the interface of the two phases and the catalytic effect of the polymerization reaction to form an insoluble film encapsulated on the surface of the UV top coating to form the wall–core structure [44]. The preparation process of UV top coating microcapsules was divided into three steps. The preparation of 1# UV top coating microcapsules was taken as an example, where # represents the unit of sample number:

Preparation of the wall materials: According to the molar ratio of 3.5:1.0, 13.52 g formaldehyde resin with a concentration of 37% and 6.00 g melamine were weighed in a beaker. Then, 30.00 mL of deionized water was added to the beaker to prepare a solution. The pH of the solution was adjusted to about 9.0 by using triethanolamine. The beaker was placed in a water bath. The temperature of the water bath was adjusted to 60 °C; the rotation speed was 700 rpm. The reaction was carried out for 20 min to obtain the solution of wall materials, which was kept warm and set aside.

Preparation of the core material: First, 0.08 g of Triton X-100 and 0.22 g of Span 20 were weighed in a beaker. Then, 78.90 mL of ethanol was added, and an emulsifier solution was obtained by thorough stirring, and then 8.80 g of the UV top coating was added. The beaker was placed in the water bath, the temperature was adjusted to 60 °C, the rotation speed was 700 rpm, and the reaction was carried out for 70 min to obtain the emulsion of core materials [45].

Preparation of UV top coating microcapsules: The solution of wall materials was gradually added to the emulsion of core materials and ultrasonicated for 15 min. After ultrasonication was completed, the mixed emulsion was placed back into the water bath, and the pH of the mixed emulsion was adjusted to about 4.0 by using the citric acid monohydrate. After filtration, the obtained powder was dried in an oven at 60 °C. The 1# UV top coating microcapsules were prepared as a result of drying. In the same way, the 2# UV top coating microcapsules and 3# UV top coating microcapsules were prepared.

### 2.3. Preparation Method of UV Coatings on the Fiberboard Surface

Fiberboard was coated manually by the method of two primers and two top coatings; i.e., two layers of primers and two layers of top coatings were applied to the fiberboard surface in turn [46,47,48]. The specific process was as follows:

First of all, after sanding with 800# sandpaper on the fiberboard surface, the surface was smooth and flat. Subsequently, a brush was used to clean up chips that had accumulated during the sanding process. Secondly, a dropper was used to add 0.40 g of UV primer on the fiberboard surface, and then a brush was used to thoroughly coat the fiberboard surface with the primer. After the coating was completed, the fiberboard was left for 1 min to make the surface coating naturally level. The fiberboard was placed on a conveyor belt of a single-lamp curing machine. The convey speed was adjusted to 0.05 m/s, and the curing time was 20 s. After curing, the fiberboard was left for 1 min to bring the coating surface down to room temperature. Then the second sanding, coating, and curing were undertaken.

After the two primers were coated, the 1#, 2#, and 3# UV top coating microcapsules were added to the UV top coatings at percentages by weight of 2.0%, 4.0%, 6.0%, 8.0%, and 10.0%. The top coating without any microcapsules was the blank control group. The total mass of each coating group was controlled to be 0.80 g. The coatings were mixed thoroughly and then coated with two top coatings according to the above primer coating method. The details of the coating ratios with different contents of the UV top coating microcapsules are shown in Table 4.

### 2.4. Testing and Characterization

#### 2.4.1. Microscopic Characterization

Scanning electron microscope (SEM): Microcapsules were prepared, and a small piece of sample was cut off perpendicular from the fiberboard surface. They were fixed to a sample plate with a double-sided adhesive for gold spraying. After gold spraying, the samples were placed on an observatory inside the SEM to carry out a vacuum operation and then adjusted to a suitable magnification for observation after a gas pressure condition was met [49,50].

#### 2.4.2. Chemical Composition

The sample was mixed thoroughly with KBr powder. The samples were then pressed into thin sheets using a powder tablet press. The chemical composition of the UV coatings on the fiberboard surface was analyzed by an infrared spectrometer.

#### 2.4.3. Optical Properties

Chromatic aberration: According to the standard GB/T 11186.3-1989 [51], a chromatic aberration meter was used to test the UV top coating. The *L* value represented the lightness and darkness value of the sample, the *a* value represented the reddish green value of the sample, and the *b* value represented the yellowish blue value of the sample. The data for the control group were recorded as *L*_1_, *a*_1_, *b*_1_, and the test data for the UV coatings with UV top coating microcapsules were recorded as *L*_2_, *a*_2_, *b*_2_. The color difference Δ*E* was calculated according to Formula (1), where Δ*L* = *L*_2_ − *L*_1_, Δ*a* = *a*_2_ − *a*_1_, Δ*b* = *b*_2_ − *b*_1_.

Δ*E* = [(Δ*L*)^2^ + (Δ*a*)^2^ + (Δ*b*)^2^] ^1/2^ (1)

Gloss: According to the standard GB/T 4893.6-2013 [52], a gloss meter was used to test the gloss degree of the UV coatings prepared with different contents of UV top coating microcapsules. The gloss degree of the UV coating was tested and recorded at three angles of incidence (20°, 60°, and 85°) to compare the differences. The gloss was the reflectivity of a surface, i.e., the brightness of the coating surface, usually expressed as a percentage. The value at 60° was used to compare gloss grades and to classify gloss categories.

Reflectance: The coating reflectance in the visible wavelength range was tested using a UV spectrophotometer. Reflectance refers to the ratio of the intensity of the reflected light to the intensity of the incident light when a beam of light strikes an object and is often expressed as a percentage [53].

#### 2.4.4. Mechanical Properties

Impact resistance: According to GB/T 1732-2020 [54], the impact resistance properties of the coating were tested. The fiberboard samples were placed on a horizontal base. A steel ball was lifted to the specified impact height (50 mm) for testing. The impacts were applied to 5 parts of coatings on the fiberboard surfaces. After the impact was completed, the samples were observed under natural light to check the damage degree of each part. The impact resistance of the samples was rated according to the average of the damage degree, which was classified into grades 1–5, and the larger the grade number, the poorer the impact resistance of the samples.

Hardness: According to GB/T 6739-2022 [55], the coating hardness was tested. A pencil was sharpened out of the standard cross-section at one end and inserted into the test instrument. The instrument was pushed slowly and evenly on the surface of the coating. After the instrument was pushed for a certain distance, the surface condition of the coating was observed under natural light. The test was repeated by replacing the pencil with a pencil with a different hardness. The hardness of the hardest pencil that did not cause defects on the coating surface was used to indicate the hardness of the coating [56].

Adhesion: According to the standard GB/T 4893.4-2013 [57], the coating adhesion was tested. The samples were placed on the horizontal base, and the coating was cut perpendicular to the samples’ surface using a multi-blade cutting tool. The UV coatings were cut slowly and uniformly down to the fiberboard surface, and then a second cut was made in the perpendicular direction of the first cut line, eventually forming a mesh scratch. The test was performed three times. The cut area was taped with clear tape, and the adhesion grade of the samples was rated according to the average of the three results. The adhesion grade ranges from 0 to 5, and the larger the grade number, the worse the adhesion of the samples.

Roughness: A roughness meter was used to test and record the values in order to derive the trend of roughness between different UV coatings.

#### 2.4.5. Self-Healing Properties Test

A blade was used to make a scratch of about 15 mm in length on the coating of the fiberboard surface. The treated fiberboard was placed on the observatory of an optical microscope and illuminated from a fixed angle using an external light source. The width of the widest part of the crack was observed and measured by the supporting software. The data were recorded as *W*_1_. After one week, the secondary width measurement was carried out at the same position of the fiberboard crack, and the width data were recorded as *W*_2_. The self-healing rate of the surface coating of the fiberboard (*W*) was calculated as shown in Equation (2). The self-healing rate was used to compare the difference in self-healing properties among different coatings of the fiberboard surface.

*W* = [(*W*_1_ − *W*_2_)/*W*_1_] × 100% (2)

These tests were repeated four times, and the error margins were controlled to be less than 5%.

## 3. Results and Discussion

### 3.1. Macroscopic Analysis of the UV Coatings on Fiberboard Surfaces

**Figure 1:**
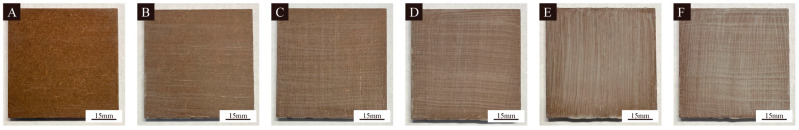
Surface morphology of fiberboard with different contents of 1# microcapsules added: (**A**) 0%, (**B**) 2.0%, (**C**) 4.0%, (**D**) 6.0%, (**E**) 8.0%, (**F**) 10.0%.

**Figure 2:**
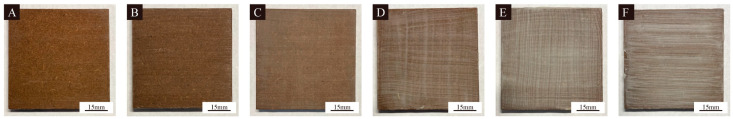
Surface morphology of fiberboard with different contents of 2# microcapsules added: (**A**) 0%, (**B**) 2.0%, (**C**) 4.0%, (**D**) 6.0%, (**E**) 8.0%, (**F**) 10.0%.

**Figure 3:**
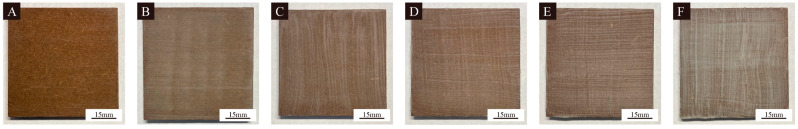
Surface morphology of fiberboard with different contents of 3# microcapsules added: (**A**) 0%, (**B**) 2.0%, (**C**) 4.0%, (**D**) 6.0%, (**E**) 8.0%, (**F**) 10.0%.

The coating morphology on the fiberboard surface with 1#, 2#, and 3# UV top coating microcapsules with different contents is shown in Figure 1, Figure 2 and Figure 3. As can be seen from Figure 1A, because the UV top coating itself was white, even the color of the coatings on the fiberboard surface in the blank control group without microcapsules was white compared to the fiberboard itself. When the content of UV top coating microcapsules was more than 6.0%, the coating on the fiberboard surface gradually appeared as a mesh pattern. This is because when the UV top coating microcapsules in the powder state are mixed with the UV top coating, the liquidity of the coating gradually becomes weaker with the gradual increase in the content of the UV top coating microcapsules. This led to an uneven dispersion of the microcapsules in the coating. Then, the UV top coating microcapsules were further aggregated in the direction of the coating process, and two layers of the top coating were stacked to form a meshed bulge structure.

**Figure 4:**
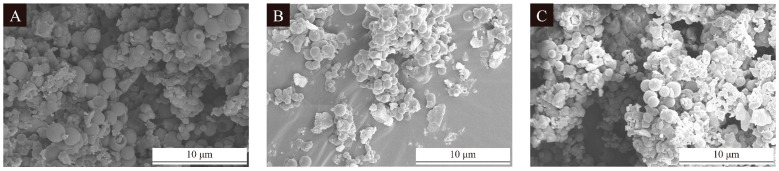
SEM images of UV top coating microcapsules: (**A**) 1# microcapsule, (**B**) 2# microcapsule, (**C**) 3# microcapsule.

**Figure 5:**
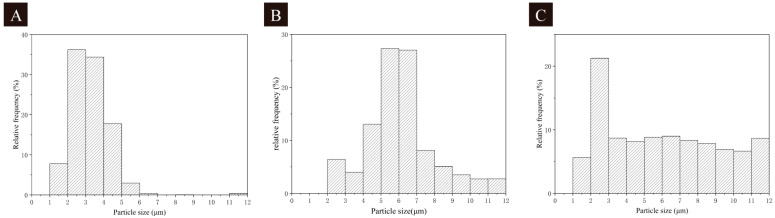
Particle size analysis images of UV top coating microcapsules: (**A**) 1# microcapsule, (**B**) 2# microcapsule, (**C**) 3# microcapsule.

The SEM images of the microcapsules are shown in Figure 4. The SEM images visualized the morphology of the microcapsules, showing that the 1# microcapsules had the best morphology with less agglomeration, while the 2# and 3# microcapsules had more serious agglomeration. The particle size distribution of microcapsules is shown in Figure 5. The 1# microcapsules had the highest distribution of particle size between 2 and 5 μm, with a smaller and more stable distribution of microcapsules. The 2# microcapsules had the highest distribution of particle size between 5 and 7 μm, with a more concentrated distribution and larger particle size than that of the 1# microcapsules. The 3# microcapsules had a distribution of 2–12 μm, which indicated that some of the particle sizes of the 3# microcapsules were too large and the distribution of the particle size was more dispersed. An analysis of the SEM and particle size distribution graphs revealed that the 1# microcapsules had the best morphology, a more concentrated distribution of particle size, and a smaller particle size compared to the 2# and 3# microcapsules.

**Figure 6:**
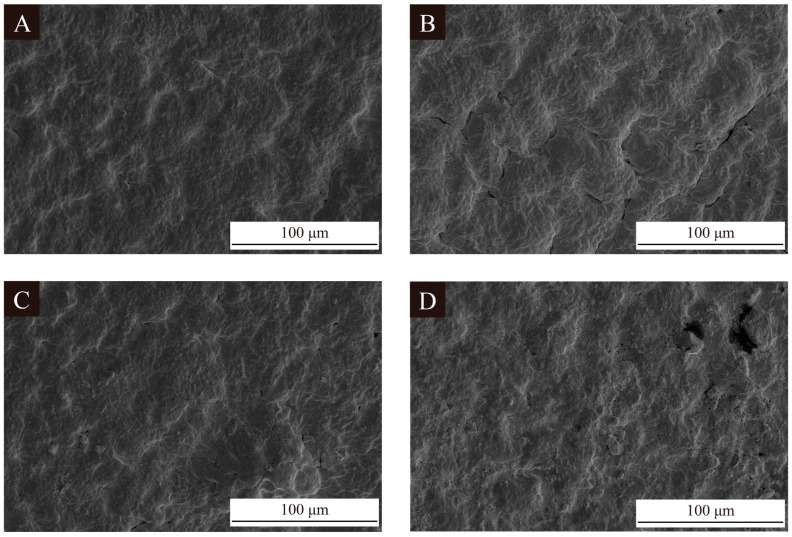
SEM images of coating on the fiberboard surface with UV top coating microcapsules: (**A**) blank control samples, (**B**) 2.0% 1# microcapsule, (**C**) 2.0% 2# microcapsule, (**D**) 2.0% 3# microcapsule.

**Figure 7:**
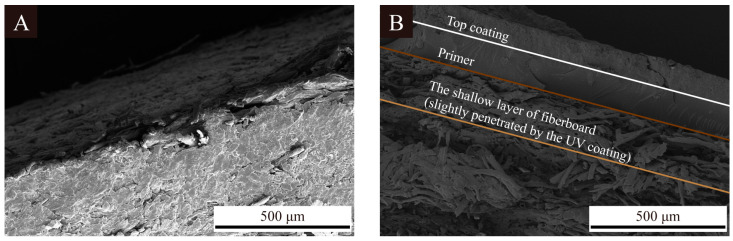
SEM image of longitudinal section of fiberboard: (**A**) blank samples, (**B**) 2.0% 1# microcapsule.

Figure 6 shows SEM images of the coatings on the fiberboard surfaces without microcapsules added and with 2.0% 1# microcapsules, 2# microcapsules, and 3# microcapsules added. The coating on the fiberboard surface without microcapsules was smooth. When the content of microcapsules was 2.0%, the coating was uneven and fish-scale-like. The coating on the fiberboard surface with 2.0% 2# and 3# UV top coating microcapsules was irregular and the whole surface was more uneven. This is because the 2# and 3# UV top coating microcapsules themselves are larger in particle size compared to the 1# UV top coating microcapsules. It was easier for the coating on the fiberboard surface to form bumps and depressions when 2# and 3# microcapsules were added to the coating, which led to a greater change in the coating morphology. Figure 7 shows the SEM image of the longitudinal section of the fiberboard coated with UV coating. Compared with the blank fiberboard, it can be seen that the coating and the longitudinal section of the fiberboard have a significant difference and are tightly coupled with each other to form a good interfacial relationship. At the same time, the penetration of the coating into the fiberboard enhanced the densification of the shallow layer of fiberboard, which is beneficial for the comprehensive utilization of the fiberboards.

### 3.2. Chemical Analysis of the UV Coating on Fiberboard Surfaces

**Figure 8:**
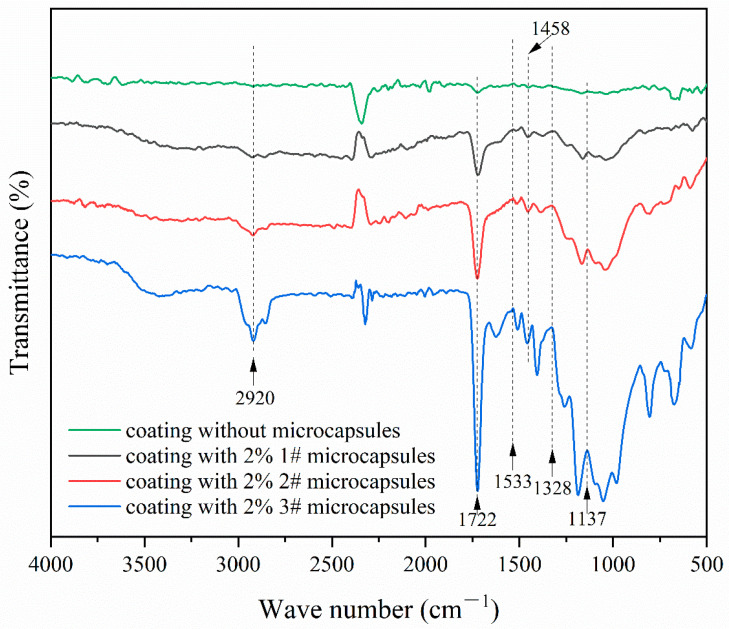
Infrared spectrum of coating on fiberboard surface.

Figure 8 shows the infrared spectra of the surface coating on fiberboard without UV top coating microcapsules and that with 1#, 2#, and 3# UV top coating microcapsules. The peaks at 1137 cm^−1^, 1328 cm^−1^, and 1533 cm^−1^ were the C-O-C stretching vibration peaks, C-N stretching vibration peaks, and N-H bending vibration peaks, respectively. The presence of these peaks in the three curves from coatings containing UV top coating microcapsules indicated that the wall materials were present and the chemical composition of wall materials remained unchanged in coatings containing UV top coating microcapsules. The bending vibration peaks of C-H contained in the core materials and UV top coatings at 1458 cm^−1^ and the C=O stretching vibration peaks and C-H stretching vibration peaks at 1722 cm^−1^ and 2920 cm^−1^ in the core materials and UV top coatings, respectively, presented in the four curves indicated that the incorporation of UV top coating microcapsules had no effect on the curing process of the UV top coatings. The UV top coating microcapsules were demonstrated to exist stably in the UV top coating.

### 3.3. Optical Analysis of the UV Coating on Fiberboard Surfaces

#### 3.3.1. Analysis of Chromatic Aberration and Chromaticity Value of the UV Coating on Fiberboard Surfaces

**Figure 9:**
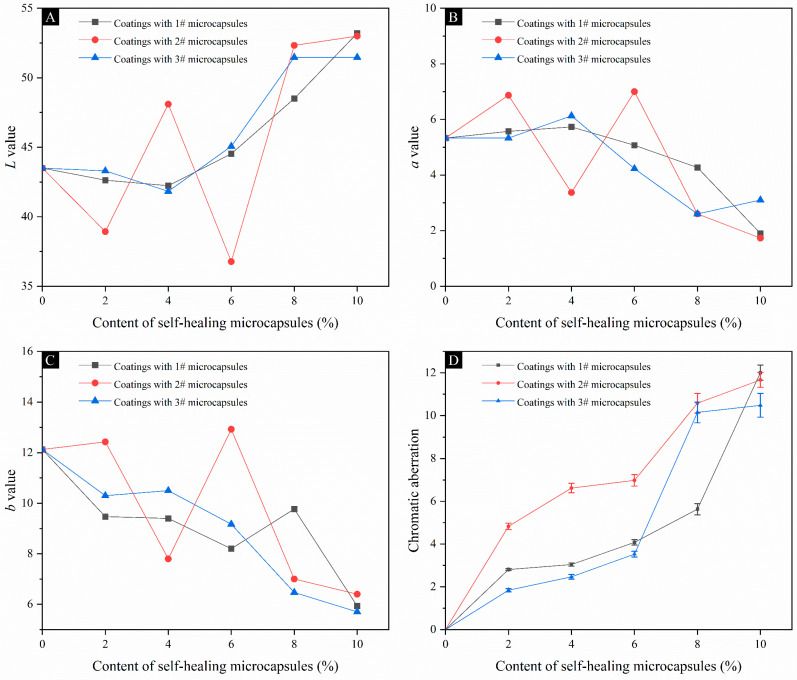
Chromaticity and chromatic aberration changes of coatings on fiberboard surface with different contents of microcapsules: (**A**) *L* value, (**B**) *a* value, (**C**) *b* value, (**D**) chromatic aberration.

Table 5 and Figure 9 show the effects of three types of UV top coatings with different contents of UV top coating microcapsules on the chromaticity values and chromatic aberration of the coating on fiberboard surfaces. The *L* value of the coating on the fiberboard surface with the increase in the content of 1#, 2#, and 3# microcapsules showed an overall trend of decreasing and then increasing. The brightness of the UV coating on the fiberboard surface went through the process of decreasing and then increasing. At the same time, the *L* value of the UV coating on the fiberboard surface of the three groups was close to 50, which indicated that the overall color of the coating was closer to gray. This is because the color of the fiberboard itself is brown. Based on the structural characteristics of the fiberboard, there will be a slight penetration of the coating to the surface layer of the fiberboard, resulting in the deepening of the color of the surface layer and the *L* value becoming smaller. When the content of UV top coating microcapsules was gradually increased, because the coating and the microcapsules themselves were white, the color of the coating became whiter and whiter, resulting in a larger *L* value. At the same time, because of the overlay of brown and white, the overall color of the coating turned gray. The *b* value of the coating on the fiberboard surface prepared with the content of 1#, 2#, and 3# microcapsules was positive, which indicated that the color of the coating on the surface of the fiberboard was yellowish overall, which was consistent with the morphology of the fiberboard surface. The *b* value showed an overall decreasing trend with the increase in the content of the UV top coating microcapsules, which indicated that the color of the coating gradually changed from the yellow color. This is because the color of the coatings and the color of the UV top coating microcapsules have caused a weakening effect on the color of the fiberboard, which is in accordance with the analysis of the *L* value. When the content of 1# microcapsules is 10.0%, the coating chromatic aberration reach the maximum value of 12.01, which is consistent with the observation of macroscopic color on the fiberboard surface as shown in Figure 1, Figure 2 and Figure 3. The error margin of chromatic aberration values was controlled to be less than 5%.

#### 3.3.2. Gloss and Reflectance Analysis of the Coating on Fiberboard Surfaces

**Figure 10:**
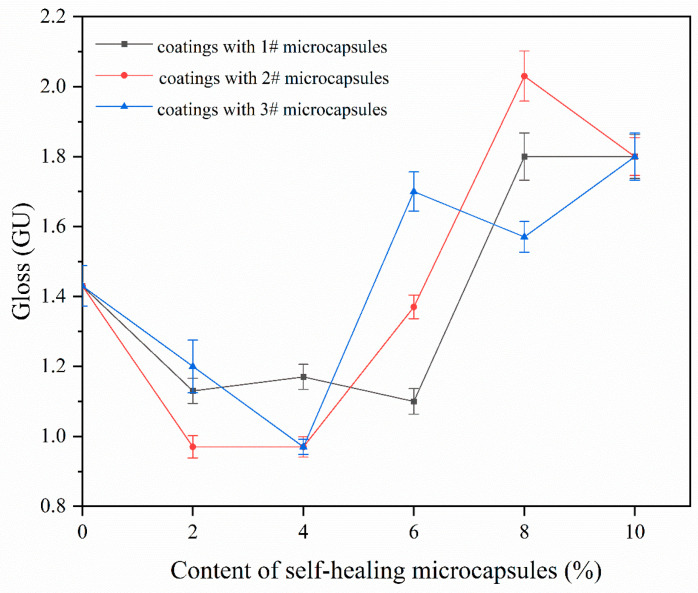
Gloss changes of coatings on the fiberboard surface with different contents of microcapsules at a 60° incidence angle.

Table 6 shows the changes in gloss and reflectance of the coating on the fiberboard surface caused by 1#, 2#, and 3# UV top coating microcapsules with different contents of the UV top coating microcapsules. Figure 10 shows the gloss of coating on the fiberboard surface at a 60° incidence angle with different contents of UV top coating microcapsules. The gloss of the coating on the fiberboard surface with the content of 1#, 2#, and 3# UV top coating microcapsules showed an overall trend of decreasing and then increasing with the increase in the content of microcapsules. The reflective properties of the coating on the fiberboard surface were weakened and then strengthened. This is because when the content of the microcapsules is small, the powdered microcapsules are dispersed in the coating in various places, which causes a change in the coating’s flatness and makes the coating increase in diffuse reflectance. As the content of microcapsules gradually increased, more microcapsules were dispersed in each area of the coating, the relative flatness increased, and the gloss increased.

**Figure 11:**
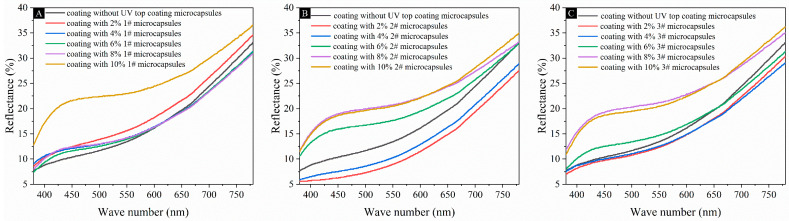
Reflectance of coatings on fiberboard surfaces with different contents of microcapsules added: (**A**) 1# microcapsules, (**B**) 2# microcapsules, (**C**) 3# microcapsules.

Figure 11 shows the reflectance of the coating on the fiberboard surface with different contents of UV top coating microcapsules. The reflectance of the coating on the fiberboard surface with 1# microcapsules increased with the increase in the content of UV top coating microcapsules. The reflectance of the coating on the fiberboard surface with 2# and 3# microcapsules increased except for a slight decrease for 2.0% and 4.0%. The trend was basically the same as that of the coating gloss degree. The reflectance of the coating on the fiberboard surface in the three groups of samples reached the maximum values of 25.11%, 22.84%, and 23.39% for the microcapsule contents of 10.0%, 10.0%, and 8.0%, respectively. The error margin of gloss and reflectance was controlled to be less than 5%.

By comprehensive comparison of the chromatic aberration, gloss, and reflectance of three group coatings on the fiberboard surface, with the increase in the content of UV top coating microcapsules, the chromatic aberration of three group samples gradually increased. The gloss first increased and then decreased, and the overall reflectance exhibited a small increase. However, the overall changes in the three parameters were small, which indicated that the different contents of UV top coating microcapsules had a small effect on the optical properties of the coating.

### 3.4. Mechanical Analysis of the UV Coating on Fiberboard Surfaces

Table 7 shows the changes in the mechanical properties of UV top coating microcapsules caused by different contents of UV top coating microcapsules on the fiberboard surface coating. The coating adhesion on the fiberboard surface with the content of 1# UV top coating microcapsules was grade 2 at all contents of UV top coating microcapsules below 4.0%. When the content increased to 6.0% the adhesion began to decrease, and the lowest decreased to grade 3. The coating adhesion on the fiberboard surface prepared with 2# UV top coating microcapsules also remained at grade 2 at 4.0% or less of the content of UV top coating microcapsules. When the content of UV top coating microcapsules on the fiberboard surface was increased to 6.0% or more, the coating adhesion began to decrease, and it decreased to grade 4 at the lowest level. The coating adhesion on the fiberboard surface with 3# UV top coating microcapsules was maintained at grade 2 when the content of UV top coating microcapsules was below 6.0%, decreased to grade 3 when it was increased to 8.0%, and decreased to grade 4 when it was increased to 10.0%, which was negatively correlated with the content of UV top coating microcapsules for all three groups of samples. Because of the spherical structure of the microcapsules, their effect on the coatings will also occur in the coating–fiberboard interface. The microcapsules added to the coating caused by the tiny curved bumps will inevitably affect the combination of the coating and the fiberboard, resulting in a decrease in the coating adhesion. The coating on the fiberboard surface with the content of 1# UV top coating microcapsules was the best compared to the other two groups because 1# microcapsules had the smallest particle size among the three types of UV top coating microcapsules and had the smallest effect on the coating adhesion.

The coating hardness of the three groups of samples was positively correlated with the content of UV top coating microcapsules, and the largest hardness reached 3H. The coating hardness on the fiberboard surface with the content of 1# microcapsules reached 3H at 2.0%, and the coating hardness on the fiberboard surface with the content of 2# microcapsules reached 3H at 8.0%. The coating hardness on the fiberboard surface with the content of 3# microcapsules reached 3H at 4.0%. The 1# UV top coating microcapsules could obtain higher coating hardness at lower content of UV top coating microcapsules, which indicated that the 1# microcapsules had the most significant enhancement effect on the coating hardness.

The impact resistance grade of the coating on the fiberboard surface of the three groups was also positively correlated with the content of UV top coating microcapsules. The impact resistance grades of the coatings on the fiberboard surface with the UV top coating microcapsules of 1#, 2#, and 3# reached the highest grade 3 at 6.0%, 8.0%, and 10.0% of the content of UV top coating microcapsules, respectively, which indicated that the 1# microcapsules had the most obvious increase in the impact resistance grades of the coatings. The changes in coating hardness and impact resistance level remain consistent in the three groups of samples because the microcapsules have increased the coating density. At the same time, the dispersed spherical microcapsules constructed a solid protective structure inside the coating with the help of UV top coating microcapsules with excellent compression resistance, so that the coating hardness and impact resistance level increased with the content of UV top coating microcapsules.

The coating roughness of the three groups was positively correlated with the content of UV top coating microcapsules. The coating roughness on the fiberboard surface with 1#, 2#, and 3# microcapsules reached the maximum of 2.211 μm, 2.847 μm, and 2.485 μm, respectively, at 10.0% content. The error margin of roughness was controlled to be less than 5%. The coating roughness with the content of 1# microcapsules had the slightest change, which was in the same situation as the adhesion change. The 1# microcapsule had the smallest size and produced the least effect.

In a comprehensive consideration of the adhesion, hardness, impact resistance grade, and roughness of the coating on the fiberboard surface, with the increase in the content of UV top coating microcapsules, the adhesion of the three groups gradually decreased, the hardness and impact resistance grade increased at the same time, and the roughness increased more. The overall coating adhesion on the fiberboard surface with the content of 1# microcapsules was the best, the hardness was the best, the impact resistance level was the highest, and the roughness was the lowest. The 1# UV top coating microcapsules were the group with the best mechanical properties among the three groups of samples.

### 3.5. Self-Healing Property Analysis of the UV Coating on Fiberboard Surfaces

**Figure 12:**
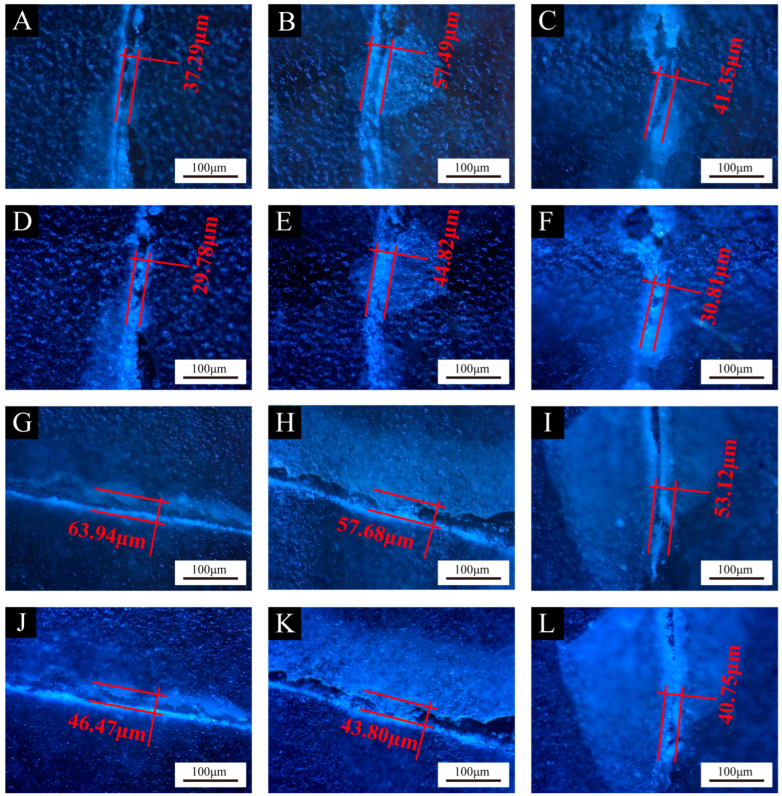
Comparison of crack width on coatings before and after 1-week self-healing with different contents of 1# microcapsules. Before self-healing: (**A**) 0%, (**B**) 2.0%, (**C**) 4.0%, (**G**) 6.0%, (**H**) 8.0%, (**I**) 10.0%; after self-healing: (**D**) 0%, (**E**) 2.0%, (**F**) 4.0%, (**J**) 6.0%, (**K**) 8.0%, (**L**) 10.0%.

**Figure 13:**
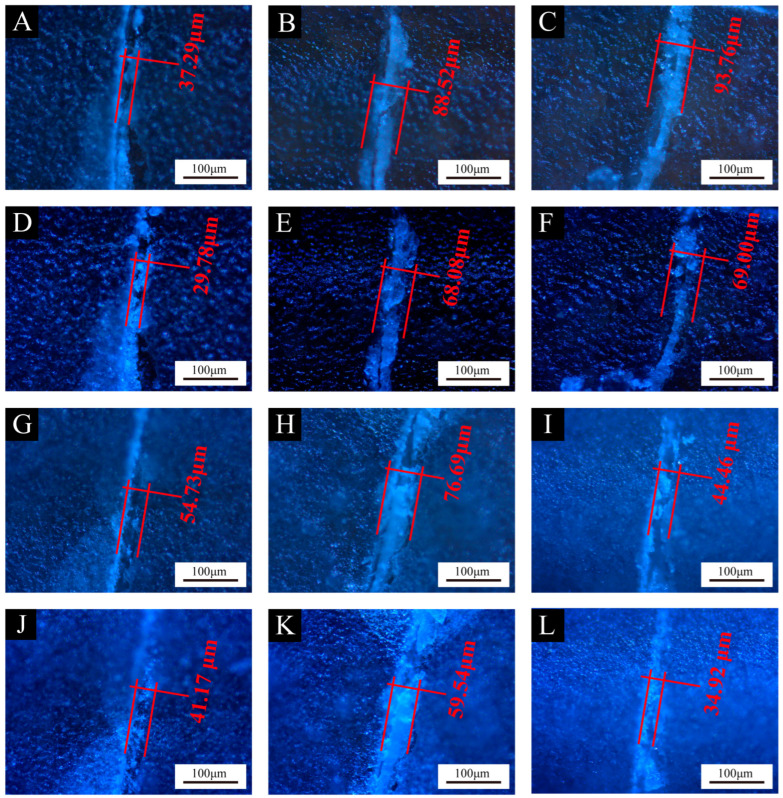
Comparison of crack width on coatings before and after 1-week self-healing with different contents of 2# microcapsules. Before self-healing: (**A**) 0%, (**B**) 2.0%, (**C**) 4.0%, (**G**) 6.0%, (**H**) 8.0%, (**I**) 10.0%; after self-healing: (**D**) 0%, (**E**) 2.0%, (**F**) 4.0%, (**J**) 6.0%, (**K**) 8.0%, (**L**) 10.0%.

**Figure 14:**
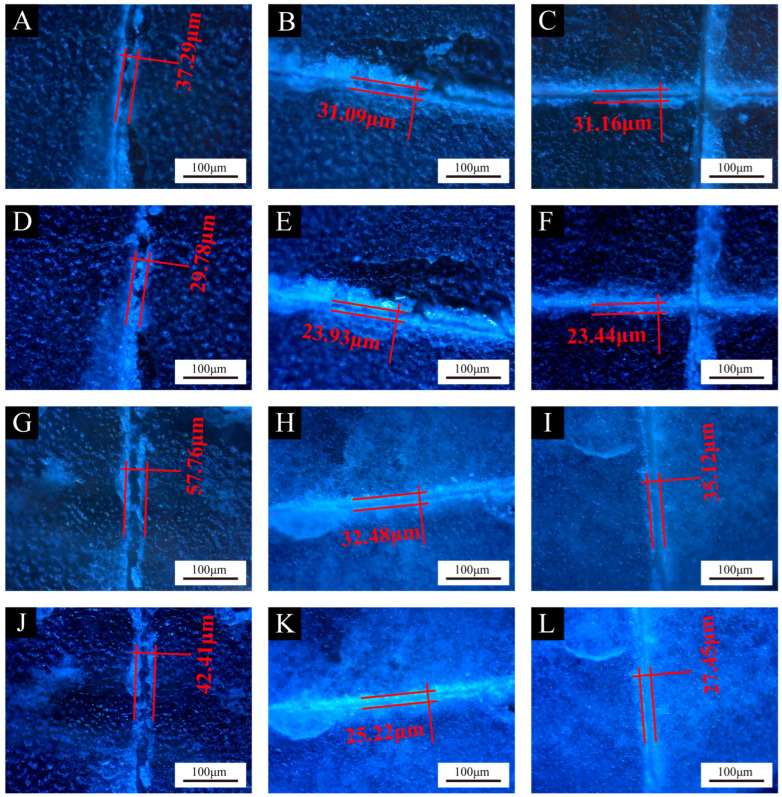
Comparison of crack width on coatings before and after 1-week self-healing with different contents of 3# microcapsules. Before self-healing: (**A**) 0%, (**B**) 2.0%, (**C**) 4.0%, (**G**) 6.0%, (**H**) 8.0%, (**I**) 10.0%; after self-healing: (**D**) 0%, (**E**) 2.0%, (**F**) 4.0%, (**J**) 6.0%, (**K**) 8.0%, (**L**) 10.0%.

**Figure 15:**
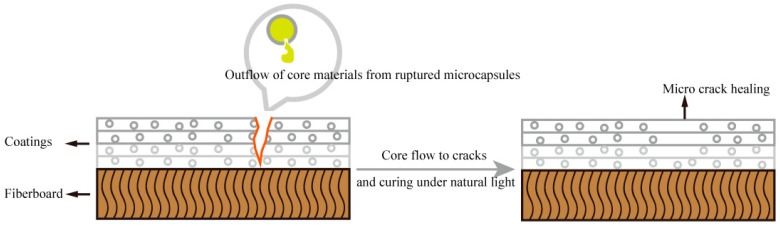
Self-healing mechanism diagram of coating on fiberboard surface.

Figure 12, Figure 13 and Figure 14 show the effect of UV top coating microcapsules on the coatings for the three UV microcapsules with different contents. The self-healing rates of UV top coating microcapsules calculated according to Figure 12, Figure 13 and Figure 14 are shown in Table 8. The self-healing rate of the coating on the fiberboard surface with 1# microcapsules at a content of 6.0% in UV top coating was the highest at 27.32%, which was 7.18% higher than the self-healing rate of the coating on the fiberboard surface without microcapsules, and the increase rate was 35.65%. The self-healing rate of coatings showed a trend of first increasing and then decreasing with the increase in the content of UV top coating microcapsules. A self-healing mechanism of the coating on the fiberboard surface is shown in Figure 15. When the coating is affected by external factors, the wall of the microcapsules is broken, and the wrapped core material, i.e., UV top coating, then flows out to fill a microcrack in the surrounding space. Because the core material itself is consistent with the materials of the coating in which it is located, the coating on the fiberboard surface can be slowly healed under the irradiation of natural light. When the content of UV top coating microcapsules was too large, too high a density of microcapsules caused stacking and extrusion. When part of the microcapsules ruptured, the core material could not flow out smoothly. At the same time, too many microcapsules would block the flow path of the core materials, and the outflow of the core material could not be efficiently utilized, which led to a decrease rather than an increase in the self-healing rate. The error margins of crack width and self-healing rates were controlled to be less than 5%.

## 4. Conclusions

Three types of UV top coating microcapsules, 1#, 2#, and 3#, were prepared and added to a UV top coating. UV coatings were then coated on the fiberboard surface without microcapsule in the UV primer, using a coating method of two primers and two top coatings. The morphology, chemical composition, optical properties, mechanical properties, and self-healing properties of the coating on the fiberboard surface were tested and analyzed. The results showed that the color of the coating on the fiberboard surface of the three groups with 1#, 2#, and 3# microcapsules gradually became whiter with the increase in the content of UV top coating microcapsules, and when the content of UV top coating microcapsules was greater than 6.0%, the coating on the fiberboard surface gradually appeared as a meshed bulge structure. In terms of microscopic morphology, the coating on the fiberboard surface with 1# microcapsules was relatively flat. With the increase in the content of UV top coating microcapsules, the chromatic aberration of the three groups of samples gradually increased, the gloss first decreased and then increased, the reflectance increased slightly, and the overall trend of optical properties was close. At the same time, the adhesion of the three groups of samples gradually decreased, the hardness and impact resistance levels increased at the same time, the roughness gradually increased, and the self-healing properties showed a trend of first increasing and then decreasing with the increase in the content of UV top coating microcapsules in the three groups of samples. The UV coating on the fiberboard surface prepared with the 1# UV top coating microcapsules in content of 6.0% in the UV top coating without microcapsules in the UV primer had better comprehensive properties, with the chromatic aberration Δ*E* of the coating of 4.08, the gloss of 1.10 GU, the reflectance of 17.13%, the adhesion of grade 3, the hardness of 3H, the impact resistance of grade 3 under the impact height of 50 mm, and the roughness of 1.677 μm. The self-healing rate of the UV coating on the fiberboard surface prepared with the 1# UV top coating microcapsules in content of 6.0% in the UV top coating without microcapsules in the UV primer was 27.32%, which was 7.18% higher than the self-healing rate of the coating on the fiberboard surface without the UV top coating microcapsules, with an increase of 35.65%. The error margins of these tests were controlled to be less than 5%. It was found that the content of microcapsules should be less; when the content of microcapsules was more than 8.0%, the microcapsules would seriously decrease the comprehensive properties of the coating and even make the self-healing rate of the coating decrease. In the future, oriented microcapsules can be considered to improve the problem of a high density of microcapsules leading to the flow obstruction of the core material toward the microcracks.

## Figures and Tables

**Table 1:** Test materials.

Test Materials	Molecular Mass (g/mol)	CAS	Producer
37% formaldehyde	30.03	50-00-0	Shandong Xinjiuchenghuagong Technology Co., Ltd., Jinan, China
Melamine	126.12	108-78-1	Jiangning District Wanjuyi Experimental Equipment Firm, Nanjing, China
Triethanolamine	149.19	102-71-6	Nanjing Houxin Biotechnology Co., Ltd., Nanjing, China
Span-20	346.459	133-39-2	Nanjing Houxin Biotechnology Co., Ltd., Nanjing, China
Triton X-100	646.85	9002-93-1	Shandong Yousuo Chemical Technology Co., Ltd., Linyi, China
Absolute ethanol	46.07	64-17-5	Wuxi Jingke Chemical Co., Ltd., Wuxi, China
UV top coating	-	-	Jiangsu Himonia Technology Co., Ltd., Zhenjiang, China
Citric acid monohydrate	210.139	5949-29-1	Jinan Xiaoshi Chemical Co., Ltd., Jinan, China

**Table 2:** Test instruments.

Test Instruments	Model	Manufacturer
Water bath	LC-OB-5L	Hunan Yunyihui E-commerce Co., Ltd., Changsha, China
Scanning electron microscope (SEM)	OLS3000	Jiangdong Jiecheng Electronic Components Store, Ningbo, China
Powder tablet press	HY-12	Tianjin Tianguang Optical Instrument Co., Ltd., Tianjin, China
Infrared spectrometer	Cary630	Shenyang Jasco Trading Co., Ltd., Shenyang, China
Chromatic aberration instrument	3nhYS3010	Shenzhen threenh Technology Co., Ltd., Shenzhen, China
Gloss meter	3nhYG60S	Shenzhen threenh Technology Co., Ltd., Shenzhen, China
Ultraviolet spectrophotometer	U-3900	Hitachi High-Tech Co., Ltd., Beijing, China
Coating roughness tester	SJ-411	Dongguan Asktools Co., Ltd., Dongguan, China
Single-lamp curing machine	620#	Huzhou Tongxu Machinery Equipment Co., Ltd., Huzhou, China

**Table 3:** A detailed list of test materials.

Sample (#)	Triton X-100 (g)	Span 20 (g)	Ethanol (mL)	UV Topcoat (g)	Formaldehyde (g)	Melamine (g)	Deionized Water (mL)
1	0.08	0.22	78.90	8.80	13.52	6.00	30.00
2	0.15	0.15	78.90	8.80	13.52	6.00	30.00
3	0.20	0.10	78.90	8.80	13.52	6.00	30.00

**Table 4:** Materials table of blend top coating.

Content of UV Top Coating Microcapsules (%)	Mass of UV Top Coating Microcapsules (g)	Mass of UV Top Coating (g)
0	0	0.800
2.0	0.016	0.784
4.0	0.032	0.768
6.0	0.048	0.752
8.0	0.064	0.736
10.0	0.080	0.720

**Table 5:** Chromaticity value and chromatic aberration of coatings on fiberboard surface with different contents of microcapsules.

UV Coatings	Content of UV Top Coating Microcapsules (%)	*L*	*a*	*b*	Δ*E*
Coatings with 1# microcapsules	0	43.50	5.33	12.13	-
	2.0	42.63	5.57	9.47	2.81
	4.0	42.23	5.73	9.40	3.04
	6.0	44.53	5.07	8.20	4.08
	8.0	48.50	4.27	9.77	5.63
	10.0	53.20	1.90	5.93	12.01
Coatings with 2# microcapsules	0	43.50	5.33	12.13	-
	2.0	38.93	6.87	12.43	4.83
	4.0	48.10	3.37	7.80	6.62
	6.0	36.77	7.00	12.93	6.98
	8.0	52.33	2.60	7.00	10.58
	10.0	53.00	1.73	6.40	11.67
Coatings with 3# microcapsules	0	43.50	5.33	12.13	-
	2.0	43.30	5.33	10.30	1.84
	4.0	41.83	6.13	10.50	2.47
	6.0	45.07	4.23	9.17	3.53
	8.0	51.47	2.60	6.47	10.15
	10.0	51.47	3.10	5.70	10.48

**Table 6:** Changes in glossiness and reflectance of coatings on the fiberboard surface with different contents of UV top coating microcapsules.

Coatings	Content of Self-Healing Microcapsules (%)	Gloss (GU)			Reflectance (%)
		20°	60°	85°	
Coatings with 1# microcapsules	0	0.20	1.43	5.13	17.10
	2.0	0.20	1.13	3.90	19.01
	4.0	0.20	1.17	3.10	17.45
	6.0	0.20	1.10	4.50	17.13
	8.0	0.23	1.80	7.00	17.34
	10.0	0.33	1.80	2.00	25.11
Coatings with 2# microcapsules	0	0.20	1.43	5.13	17.10
	2.0	0.10	0.97	3.70	12.59
	4.0	0.20	0.97	5.00	13.89
	6.0	0.30	1.37	4.93	20.37
	8.0	0.33	2.03	3.53	22.68
	10.0	0.37	1.80	8.67	22.84
Coatings with 3# microcapsules	0	0.20	1.43	5.13	17.10
	2.0	0.20	1.20	2.77	15.72
	4.0	0.20	0.97	3.37	15.67
	6.0	0.20	1.70	8.93	17.71
	8.0	0.30	1.57	2.87	23.39
	10.0	0.33	1.80	2.30	23.17

**Table 7:** Mechanical properties of coatings on fiberboard surface with different contents of microcapsules added.

Coatings	Content of Microcapsules (%)	Adhesion (Grade)	Hardness	Impact Resistance (Grade)	Roughness (μm)
Coatings with 1# microcapsules	0	2	2H	5	1.410
	2.0	2	3H	4	1.493
	4.0	2	3H	4	1.443
	6.0	3	3H	3	1.677
	8.0	3	3H	3	1.867
	10.0	3	3H	3	2.211
Coatings with 2# microcapsules	0	2	2H	5	1.410
	2.0	2	2H	4	1.507
	4.0	2	2H	4	1.669
	6.0	3	2H	4	1.927
	8.0	4	3H	3	2.248
	10.0	4	3H	3	2.847
Coatings with 3# microcapsules	0	2	2H	5	1.410
	2.0	2	2H	4	1.110
	4.0	2	3H	4	1.254
	6.0	2	3H	4	1.821
	8.0	3	3H	4	2.180
	10.0	4	3H	3	2.485

**Table 8:** Self-healing rates of coatings on fiberboard surface with different contents of microcapsules.

Coatings	Content of Microcapsules (%)	Crack Width after Scratch (μm)	Crack Width after 1-Week Self-Healing (μm)	Self-Healing Rates (%)
Coatings with 1# microcapsules	0	37.29	29.78	20.14
	2.0	57.49	44.82	22.03
	4.0	41.35	30.81	25.49
	6.0	63.94	46.47	27.32
	8.0	57.68	43.80	24.07
	10.0	53.12	40.75	23.28
Coatings with 2# microcapsules	0	37.29	29.78	20.14
	2.0	88.52	68.08	23.09
	4.0	93.76	69.00	26.41
	6.0	54.73	41.17	24.78
	8.0	76.69	59.54	22.36
	10.0	44.46	34.92	21.45
Coatings with 3# microcapsules	0	37.29	29.78	20.14
	2.0	31.09	23.93	23.02
	4.0	31.16	23.44	24.79
	6.0	57.76	42.41	26.58
	8.0	32.48	25.22	22.35
	10.0	35.12	27.45	21.83

## Data Availability

Data are contained within the article.
